# Investment case for two-year post university speciality training in family medicine in Tajikistan: how much is needed for continuing and scaling up the improved education of family doctors?

**DOI:** 10.1186/s12913-020-05953-5

**Published:** 2020-12-09

**Authors:** Jari Kempers, Leah F. Bohle, Alexandra Topa, Greta Ross, Zukhra Kasymova, Shakhlo Yarbaeva, Cristina Rotaru, Helen Prytherch

**Affiliations:** 1 Qalys Health Economics, Amsterdam, the Netherlands; 2 Swiss Tropical and Public Health Institute, Basel, Switzerland; 3 University of Basel, Basel, Switzerland; 4 State University of Medicine and Pharmacy “Nicolae Testemitanu”, Chișinău, Republic of Moldova; 5 Swiss Agency for Development & Cooperation’s Medical Education Reform Project, Dushanbe, Republic of Tajikistan; 6 Independent statistician, Chisinau, Moldova

**Keywords:** Family medicine, Family doctors, Postgraduate medical education, Residency, Internship, Tajikistan, Investment case, Cost, Scale-up, Evaluation

## Abstract

**Background:**

A new two-year Post University Specialty Training (PUST) programme in family medicine was introduced to improve the quality of postgraduate speciality medical education in Tajikistan. Postgraduate education of family doctors (FDs) needs to be urgently scaled up, as 38% of FD positions in Tajikistan remained unfilled in 2018. Moreover, the international financial support for the PUST programme is ending. This investment case assesses the minimum funding needed for the continuation and scale-up of PUST and establishes the rationale for the investment in the light of a recent evaluation.

**Methods:**

The costs of the programme were calculated for 2018 and a scale-up forecast made for the period 2019–2023. The impact of the scale-up on the shortage of FDs was assessed. An evaluation using a Multiple Choice Questionnaire and Objective Structured Clinical Examination (OSCE) assessed and compared theoretical knowledge, clinical skills and competencies of PUST trained and conventionally trained FDs.

**Results:**

The annual costs of the programme were US$ 228,000 in 2018. The total investment needed for scaling up PUST from 31 new FDs in 2018 to 100 FD graduates each year by 2023 was US$ 802,000.However, when the retirement of FDs and population growth are considered, the scale-up will result only in maintaining the current level of FDs working and not solve the country’s FD shortage. The PUST FDs demonstrated significantly better clinical skills than the conventionally trained interns, scoring 60 and 45% of OSCE points, respectively. Theoretical knowledge showed a similar trend; PUST FDs answered 44% and interns 38% of the questions correctly.

**Conclusions:**

The two-year PUST programme has clearly demonstrated it produces better skilled family doctors than the conventional one-year internship, albeit some enduring quality concerns do still prevail. The discontinuation of international support for PUST would be a major setback and risks potentially losing the benefits of the programme for family medicine and also other specialities. To guarantee the supply of adequately trained FDs and address the FD shortage, the PUST should be continued and scaled up. Therefore, it is essential that international support is extended and a gradual transition to sustainable national financing gets underway.

**Supplementary Information:**

The online version contains supplementary material available at 10.1186/s12913-020-05953-5.

## Background

The education of medical doctors in the Republic of Tajikistan is in the process of transitioning from a heavy focus on theoretical learning (remnants of the country’s Soviet past) to more practical clinical teaching and competency-based learning. This modernisation is in line with current effective educational practices [1]. The Ministry of Health and Social Protection of the Population of the Republic of Tajikistan (MoHSP) has identified medical education reform as a key priority within the National Health Strategy for 2010–2020 [2].

Important progress has been made to reform undergraduate education for medical students with clinical exposure now introduced from Year Four, and a practical clinical Year Six added in 2016. At the postgraduate level the progress is more limited. The conventional way of speciality training in Tajikistan, which continues to be offered, comprises one year of unstructured work experience. In order to improve the quality of postgraduate speciality education, a new alternative two-year Post University Speciality Training (PUST) residency programme focused on Family Medicine was developed in 2013 by the Swiss Agency for Development and Cooperation’s (SDC) Medical Education Reform Project (MEP). Key partners in this important undertaking were the MoHSP, Ministry of Education and Science, the Republican Clinical Centre for Family Medicine, the Post Graduate Medical Institute (PGMI), and the Tajik State Medical University. This alternative two-year PUST programme is implemented by the PGMI with the support of MEP.

The two-year PUST programme has a strong focus on improvement of practical clinical skills and competency based learning. Since September 2013 the programme has been implemented in the MEP project districts: Vose, Hamadoni, Fayzabad, Rudaki, Hissar, Tursunzade, Shahrinav, Penjikent, Devashtich (Ganchi), Istaravshan, and Dushanbe. PUST has two main components: 1) a theoretical part (20%) taught at PGMI or at peripheral clinical training bases, and 2) a practical training and clinical teaching part (80%) in polyclinics and rural health centres, delivered by trained and certified family doctor (FD) tutors. These newly qualified graduates who enrol in the PUST programme are referred to as *FD residents* in alignment with international practice and to distinguish them from the conventional one-year interns. The FD residents are integrated into the primary healthcare team with growing responsibility over the course of the two years. Of the 105 PUST FDs graduating between 2013 and 2018, 97% were then deployed by MoHSP and continue to work as FDs [3, 4].

According to MoHSP, 38% of the 5333 FD positions in Tajikistan were not filled in 2018 [5, 6]. Postgraduate education to provide adequately trained FDs, therefore, needs to be urgently scaled up. International financial support from the SDC is, however, coming to an end, which poses challenges for the continuity of the PUST programme and its necessary scale-up. In order to advocate for continued and sufficient funding for the PUST programme, the total cost of running the programme needed to be estimated. In 2018, PUST operated with a mixture of national and donor funding, and support received in kind from MEP. To promote a gradual transition to sustainable national financing, a combined strategy of increasing national funding while gradually decreasing international support is proposed. This investment case assesses the *minimum* funding, both national and international, needed for the continuation and scale-up of the PUST programme in 2019–2023. Moreover, the paper establishes the rationale for the investment using the results of a recent evaluation, where PUST trained FDs were demonstrated to have statistically significantly better clinical skills than the conventionally trained FDs receiving one year of unstructured work experience (interns) [7].

In the literature, there are studies on the influence of the length of postgraduate education on the professional qualities of medical graduates. However, these studies are mainly conducted in Western Europe and North America, and there are no comparable studies in the former Soviet Union countries. In Western countries, extending the length of postgraduate education leads to higher levels of knowledge and skills [8, 9]. Other findings in fact suggest, from the participants’ own assessment, that a two-year residency period is too short to achieve full competence in all areas of family medicine [10].

## Methods

### PUST costs in 2018

The actual costs of the PUST programme at PGMI were calculated in 2018, which was the closest completed financial year at the time of conducting this study. In 2018 there were 68 FD (37 first year and 31 s year) residents in the PUST programme. The cost analysis was based on the 2018 financial accounting records of the MEP programme and PGMI / MoHSP. The costs were divided into four categories: 1) Resident stipends paid by MoHSP and MEP, 2) Compensation including salaries and additional compensation for tutors, trainers and coordinators, 3) Tutor training, covering initial training and refresher training of tutors, and 4) Other, including transportation costs, medical literature and medical bags for FD residents. The analyses consider only the direct costs of the PUST programme. An overview of the input parameters and their sources can be found in the additional file 1. The costs are presented from the government (payer) perspective, as undiscounted 2018 US dollars (US$). Both, undiscounted and discounted cost results, can be found in the additional file 2. The local costs in Tajikistani somoni (TJS) were converted to US$ at the exchange rate of 9.4386 [11].

### PUST scale-up

A five-year forecast from 2019 to 2023 estimated the investment needed for scaling up the PUST programme gradually from 31 new FDs in 2018 [12] to 100 per year by 2023. To keep the scale-up forecast realistic and feasible, the number of FD residents was increased gradually by recruiting more postgraduate residents each year. A target of 100 FDs per year was selected after discussions with the PGMI, based on the current situation and as a realistic goal that could potentially be achieved in the coming five years. The forecast assumes that all new FDs graduate through the PUST programme and not via the conventional one-year work experience.

### Impact of PUST on the shortage of family doctors

The potential impact of the PUST scale-up on the shortage of FDs in Tajikistan in 2019–2023 was also assessed, while taking into consideration the approaching retirement of FDs aged over 60 years and the population growth. This forecast was done by combining the number of FDs currently working in Tajikistan [5], their age distribution [13], the percentage of FDs who stopped work for reasons other than retirement [5], and the numbers of new FDs graduating from the PUST programme. The predicted number of FDs was then compared against the current norm for FD positions in Tajikistan [14], incorporating the predicted rapid population growth of 1.9% annually [15]. The norm is based on catchment population - FD ratios in rural and urban areas. While this is not an accurate need-based indicator for the number FDs, it was the best available source at the time of conducting the study. The forecast shows the number of FDs according to the norm, in the PUST scale-up and current situation during the period 2019–2023.

### Cost forecast and transition to national funding

The forecast assumed a gradual stepwise reduction of international donor support with an increase in national investment from the MoHSP to accomplish 100% national funding by 2023. Understandably, MoHSP cannot maintain the same level of expenditure that was possible with international donor support. Therefore, to ensure the financial sustainability of the PUST programme, the following cost reductions were assumed during the transition period. First, additional compensation paid by the MEP programme for tutors, trainers and coordinators was gradually reduced to zero by 2023. After this period, personnel only received their usual salaries from MoHSP. Second, financial support for tutor training was gradually reduced to zero by 2023. Thereafter, it was assumed that MoHSP will train the necessary new tutors at a cost of US$ 74 per tutor, which represents 50% of the donor-supported training costs. The training costs per tutor are low because these are group trainings and the trainer salaries are low. Third, the provision of medical bags for new first-year postgraduates ended and costs for text books and transport were minimised. Because of these cost reductions, the forecasted MoHSP budget represents the *minimum* national investment needed for taking over the financing of the PUST programme. The costs of training one FD was calculated before and after the scale-up, in 2018 and 2023, respectively.

### PUST evaluation

Finally, the results of a recent evaluation of the PUST programme are summarised to support the investment case [6]. The evaluation compared a total of 58 Tajik FDs in three different groups: i) 26 newly graduated FDs who completed the two-year PUST, ii) 20 medical graduates who had entered the first year of the PUST, and iii) eight newly graduated FDs who completed the conventional one year intern path. Even though the number of PUST FDs and 1st year PUST graduates evaluated are relatively small, the sample sizes represent 84 and 54% of graduates in the PUST programme in 2018, respectively. Finding newly graduated FDs from the one year intern path, without additional work experience, was challenging. To ensure the comparability between the groups, interns with more work experience than the one year internship needed to be excluded. Consequently, the sample remained small.

The evaluation measured clinical and managerial skills, attitudes, behaviour and communication skills in a simulated consultation through an Objective Structured Clinical Examination (OSCE). The OSCE comprised five different stations to assess history taking, examination, interpretation of laboratory results and communication skills. OSCE scenarios were developed based on internationally existing OSCEs for family doctors, and informed by a literature review. Scenarios were adapted to the Tajik context and validated by internal and external experts.

In addition, theoretical knowledge of the FDs was tested by using a 60 item Multiple Choice Questionnaire (MCQ). The development of the MCQ was based on an initial literature review and decisions on 20 tracer diseases most relevant for the Tajik context. The design of the multiple choice questions were based on recommendations by the Yale Center for Teaching and Learning [16], the University of Waterloo Centre for Teaching Excellence [17] and Considine et al., 2005 [18]. Additional pre-tested MCQ questions were chosen from a pool of questions provided by AMBOSS GmbH [19] and were adapted to the Tajik context. A total of 60 questions pertinent to 20 tracer diseases were applied. A question stem was developed with five multiple choice response options of which one was correct. For each tracer disease (e.g. heart attack) three multiple choice questions were developed to enhance the reliability and validity of the knowledge assessed. To ensure the validity in the Tajik context, MCQs were reviewed and tested for content, face and construct validity [20] by a pool of internal and external experts. Quantitative data of the PUST evaluation was analysed using Cronbach’s alpha coefficient of internal consistency and SPSSM; chi square test, Fisher exact test (if conditions for chi square were not met) and ANOVA procedure were applied. A detailed description of the methods, psychometric analysis and results of the evaluation can be found elsewhere [Bohle, LF et al. (2020). Evaluation of two-year post university speciality training in family medicine programme in Tajikistan, Forthcoming.].

## Results

**Fig. 1:**
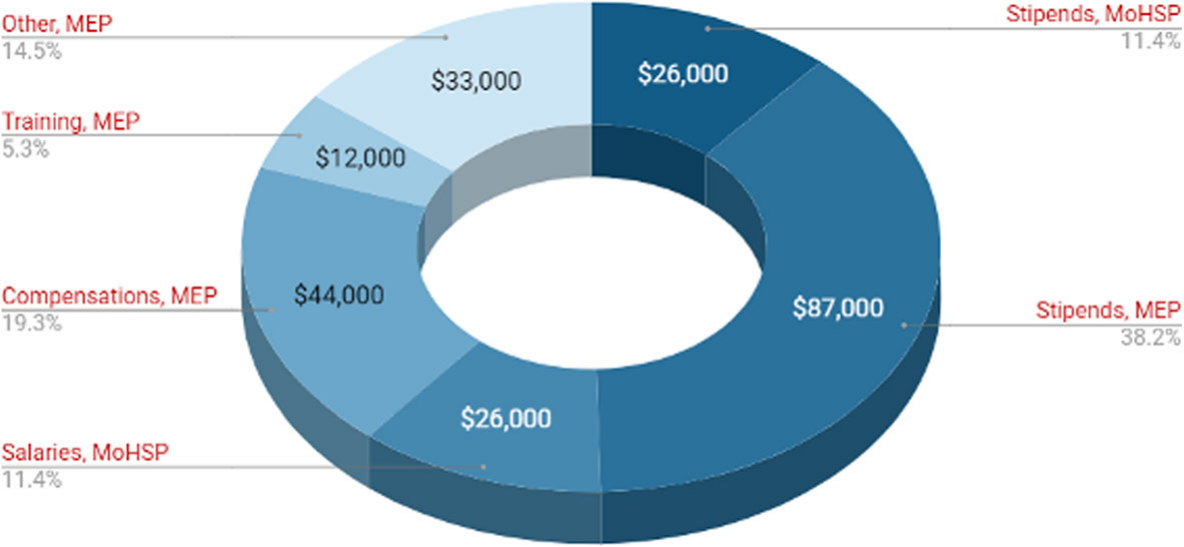
Annual costs of PUST by expense category and payer in US$ in 2018. Expense categories: Resident stipends of MoHSP and MEP. PUST related portions of salaries of tutors, trainers and coordinators. Additional compensations for tutors, trainers and coordinators financed by MEP. Tutor training, covering training and refresher training, provided by MEP. Other, including transportation costs, medical literature and medical bags for the PUST residents covered by MEP

The total annual costs of the PUST programme in 2018 were US$ 228,000. Of these, US$ 51,000 (22.4%) were financed by MoHSP and the majority US$ 177,000 (77.6%) by the international donor SDC. Figure 1 shows a breakdown of expenditure in 2018. Of the total, US$ 26,000 (11.3%) was used on MoHSP’s resident stipends and US$ 87,000 (38.1%) on MEP’s resident stipends. The PUST related portion for salaries of tutors, trainers and coordinators was US$ 26,000 (11.2%). Additional compensation paid by MEP to tutors, trainers and coordinators accounted for US$ 44,000 (19.5%). Tutor training cost US$ 12,000 (5.3%), and other costs came to US$ 33,000 (14.6%).

### Scale-up of PUST FDs

**Fig. 2:**
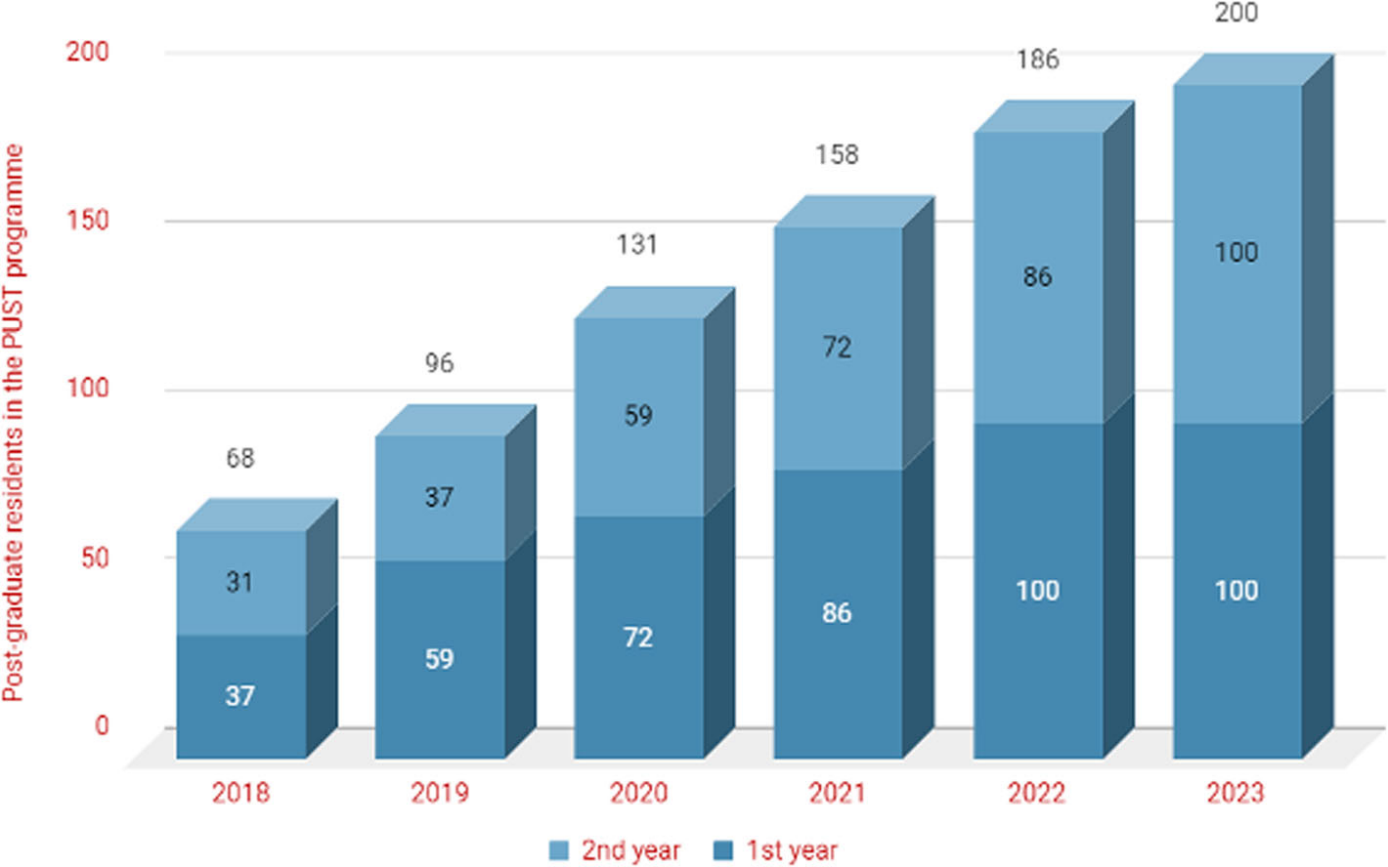
Scale-up forecast of 1st and 2nd-year residents in the PUST programme 2018–2023

Figure 2 shows the scale-up forecast for the period 2018–2023. In 2018, there were 37 first-year PUST residents and 31 who graduated as PUST FDs. In the forecast, the PUST programme is gradually scaled up from 31 FDs graduating in 2018 to 100 FDs in 2023. In order to do this, the number of first-year residents recruited into the PUST programme is increased each year. As the PUST is a two-year programme, there are two year groups, 1st and 2nd year, going through the forecast.

### Impact on the shortage of family doctors

**Fig. 3:**
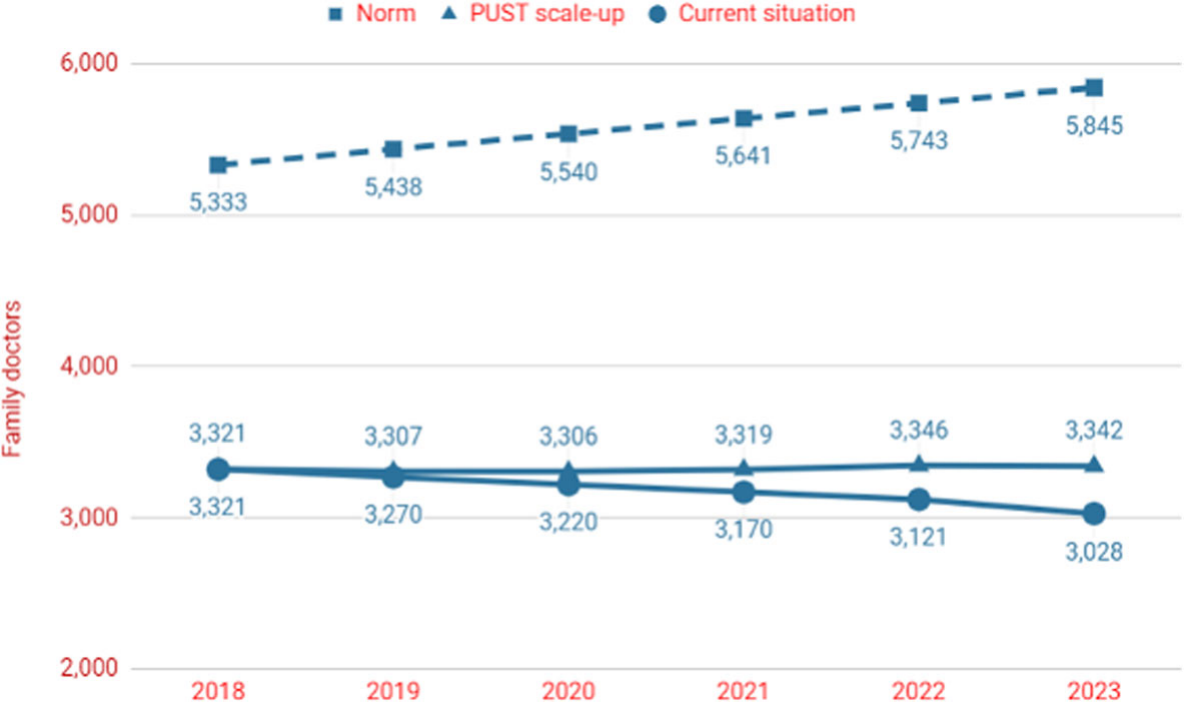
Forecasted numbers of family doctors (FDs) needed according to the norm in Tajikistan, FDs working in the PUST scale-up scenario and in the current situation

According to the current norm, there were 5333 FD positions in Tajikistan in 2018 [10]. However, MoHSP estimated that there were only 3321 FDs working in 2018 [5]. Hence, there is a gap of 2012 FDs. A significant proportion of the working FDs (36%) were in the age bracket 50–60 years, therefore approaching retirement [9]. In the next five years, 12.6% of FDs will retire, and the population of Tajikistan is projected to grow by 9.5% [11, 21]. When the population growth is factored in, the number of FDs needed increases to 5845 by 2023 (Fig. 3). If the current number of new FDs graduating is not increased, and taking the approaching retirements into account, the number of FDs working declines to 3028 by 2023 (current situation). If the PUST is scaled up to 100 graduates per year by 2023, taking into account the retirements, the number of working FDs increases only marginally to 3342 FDs by 2023, and the percentage of filled FD positions declines only slightly from 62% in 2018 to 57% in 2023. The 100 new FDs annually represent a significant scale up of the PUST programme, which will help to increase the supply of better trained FDs and maintain the current number of working FDs. However, it will not be a solution for solving Tajikistan’s FD shortage.

### PUST cost forecast and transition to national funding

**Fig. 4:**
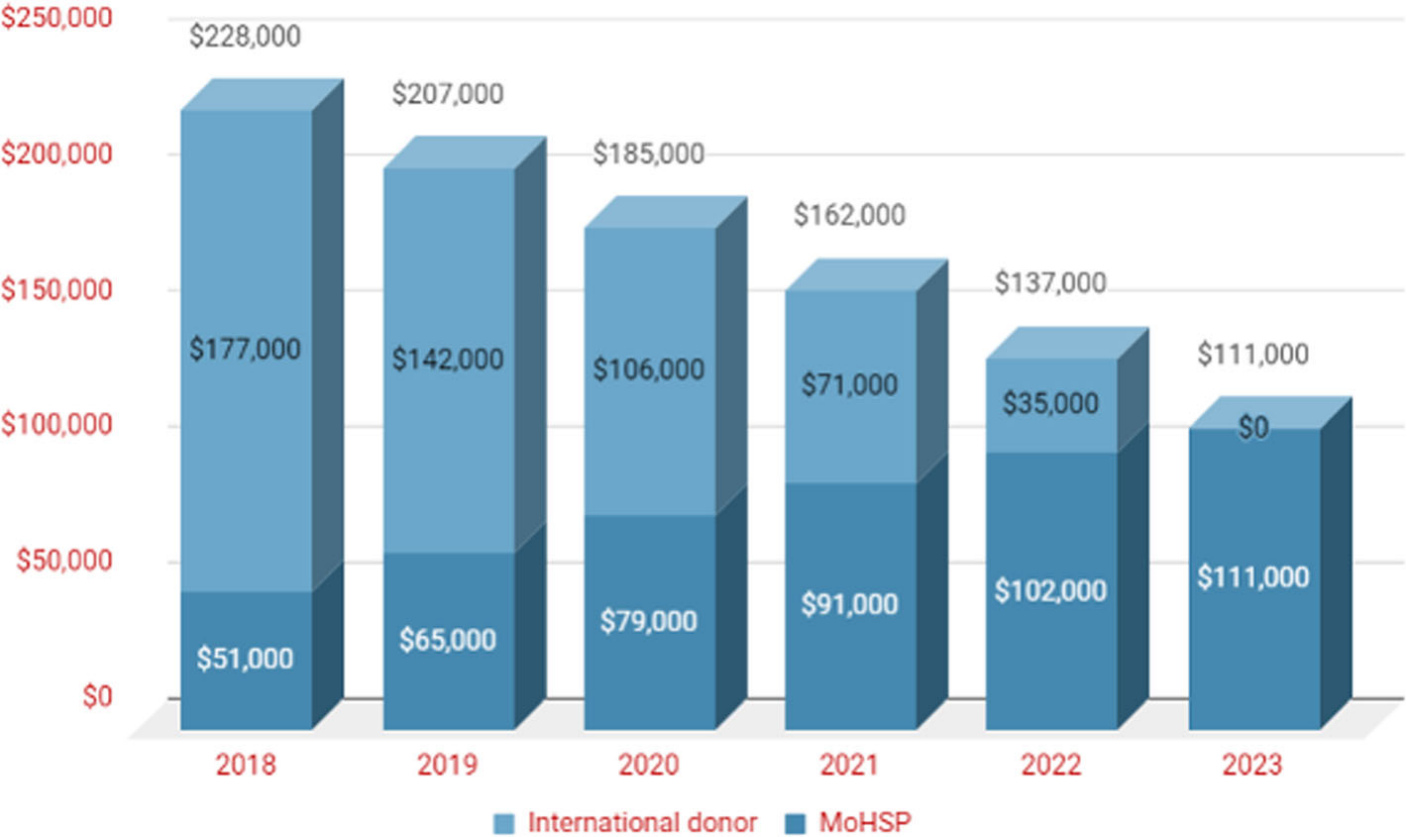
Annual budget of PUST in 2018–2023, with a gradual transition to 100% national funding in 2023

The total investment needed for the PUST programme scale-up was calculated to be US$ 802,000 for the period 2019–2023. The forecast assumed a gradual transition to 100% national funding by 2023, with the international donor support decreasing in a stepwise manner from 2018 levels to zero by 2023 (Fig. 4). Thus, the total financial support needed from an international donor for 2019–2023 was calculated to be US$ 354,000.

MoHSP cannot maintain the same level of expenditure that was possible with international donor support. Therefore, to ensure the financial sustainability of the PUST programme, the above-mentioned cost reduction measures, i.e. limiting additional compensation for personnel, stipends, medical bags and other expenses, were assumed during the transition period. Consequently, the forecast presents the *minimum* national investment needed to take over the financing of the PUST programme. The total national investment needed from MoHSP for the PUST programme was calculated to be US$ 448,000 for 2019–2023. Even though available national funds are limited, the annual budget of MoHSP will need to be increased 2.2 fold, from US$ 51,000 in 2018 to US$ 111,000 in 2023. As a result of the cost reductions and the scale increase, the cost of training one PUST FD was predicted to decline significantly from US$ 6730 in 2018 to US$ 1110 in 2023.

### The benefit of the PUST programme

**Fig. 5:**
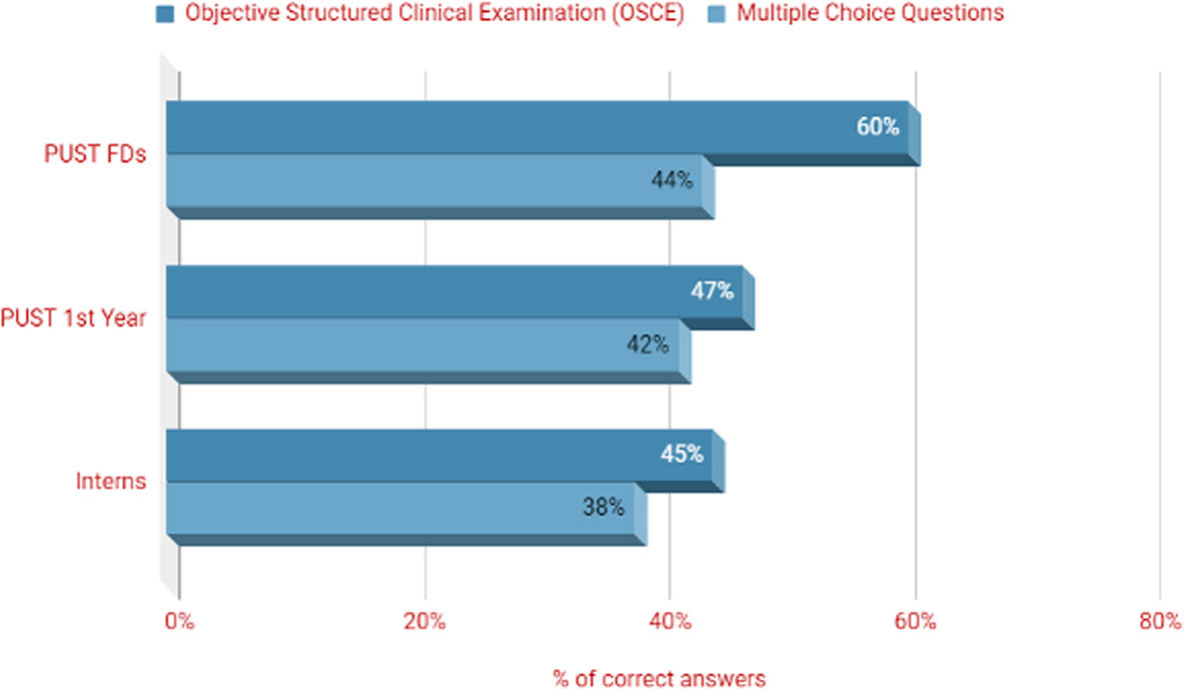
Result of PUST evaluation Objective Structured Clinical Examination (OSCE) and Multiple Choice Question scores [6]. Mean Objective Structured Clinical Examination (OSCE) scores and Multiple Choice Questions (MCQ) scores of 2nd year PUST family doctors (PUST FDs), 1st year residents entering the PUST (PUST 1st year) and newly graduated FDs who completed the conventional one-year work experience (interns)

The PUST programme was evaluated by comparing the clinical skills and competencies, attitudes and theoretical knowledge of i) newly graduated PUST FDs, ii) 1st year residents entering the PUST (PUST 1st year), and iii) new FDs who completed the conventional one year work experience intern path (interns) [6]. Overall, the PUST FDs achieved significantly better OSCE scores than the conventionally trained interns (*N* = 26 60%; versus *N* = 8 45%) (Fig. 5). Interestingly, the 1st year PUST residents also had better OSCE scores than the interns (*N* = 20 47% versus N = 8 45%). Newly graduated PUST FDs scored highest in all stations with the focus on history taking, physical examination, main diagnosis and next diagnostic steps. Communication skills were assessed in four stations. The analysis demonstrated that the newly graduated PUST FDs performed statistically significantly better than the other two groups (F = 8.64, *P* = 0.01). Interestingly, a statistically significant gender difference among 2nd year PUST FDs was also observed, with females performing better in history taking than their male colleagues.

Theoretical knowledge assessed by the MCQ questionnaire showed a similar trend, albeit with less variation between scores. Overall the reliability of the MCQ scores presented here, using Cronbach’s alpha coefficient of internal consistency, is 0.87. The estimated reliability is hence adequate for research purposes such as correlational analysis and means comparisons [22, 23]. Out of the 60-item MCQ questions, PUST FDs answered 44% correctly, followed by 1st-year PUST residents (scoring 42%) and interns (scoring 38%) (Fig. 5). The 2-year PUST FDs performed significantly better compared to PUST 1st year participants (using the Pearson Chi-Square test) on eight important tracer diseases and differential diagnoses, including acute earache (χ^2^ = 13.967 *p* = .001), breast cancer (χ^2^ = 13.405p = .001), iodine deficiency groups (χ^2^ = 7.875 *p* = .019), and difficulty in breathing (χ^2^ = 7.663 *p* = .022). The results demonstrate that the 2-year PUST FDs performed best in both tests. The combined final scores of the two tests (OSCE and MCQ) of the PUST FDs (t = 5.798; *p* < 0.001) and PUST 1st year (t = 2.262, *p* = 0.036) are statistically significantly better than those of the interns. These findings highlight the achievements and benefits of PUST, especially regarding improvements in clinical skills for FDs starting their career. The results also indicate a lack of professional progression in knowledge and clinical skills by the interns going through the conventional one-year internship.

## Discussion

Financing of the two-year Post University Specialty Training (PUST) in Family Medicine should be continued and the programme scaled up, as it has clearly demonstrated to produce better skilled family doctors than the conventional one-year internship. The ending of the SDC donor support in 2019 is likely to pose significant challenges to the quality, continuation and the needed scale-up of PUST. Therefore, it is essential that international financial support for PUST is continued to ensure the continuity of the training and a structured transition to sustainable national funding.

In the next five years (2019–2023), a total investment of US$ 802,000 is needed to continue and to scale up the PUST programme so that 100 new FDs graduate annually by 2023. Of this, US$ 355,000 is needed from an international donor. The minimum investment needed from MoHSP is US$ 448,000. Importantly, the actual budget impact on MoHSP is lower than this, as it is already paying a part of the PUST FDs’ stipends, and all salaries of tutors, trainers and coordinators. It is recommended that the international funding is gradually decreased to push and prepare for the transition to full national funding. In parallel, donor financing should be made conditional on the increase of national funding and the scale-up of FDs graduating from the programme. According to this research, the cost reductions and the scale increase contribute to reducing the cost of training for one PUST FD significantly, from US$ 6730 in 2018 to US$ 1110 in 2023. This suggests a more efficient use of limited resources.

The current number of new FDs graduating is too low to maintain the current coverage of 62% of FD positions being filled, especially considering the approaching retirement of FDs aged over 60 and the population growth. In 2018, approximately 60 new FDs graduated: 30 PUST FDs and 30 interns. The PUST programme needs to be urgently scaled up. However, even scaling up to 100 new PUST FDs graduating each year will only maintain the current number of working FDs and it does not solve the chronic shortage of FDs. At least 200 new FDs should graduate annually to start closing the FD gap. However, in the current situation with funding and capacity challenges, and limited interest in Family Medicine among graduating medical doctors, scaling up to PUST to 200 FDs per year is not a feasible and realistic target.

The results of the PUST evaluation clearly demonstrate that PUST FDs have better clinical skills than the interns [6]. This is in keeping with results obtained in other contexts where competency-based learning was introduced [24]. Moreover, the theoretical knowledge and clinical skills of interns did not appear to improve from levels achieved in undergraduate medical education. This suggests a lack of professional progress reflecting the limitations of the conventional one-year work experience received by interns. In light of these results, it is recommended that the PUST programme be used for future training of all FDs.

The transition to national funding provides MoHSP with an opportunity to institutionalise the PUST programme at PGMI. Moreover, a longer period of tutor-supervised practical training is one of the preconditions for international accreditation of Family Medicine education and diplomas, as called for by the World Federation of Medical Education [25]. The current PUST two-year practical training might still not be sufficient for such accreditation, but it is an important step in that direction.

International financial and technical support has been essential for the development and initiation of the PUST programme. The discontinuation of international support would be a major setback and risks potentially losing the benefits to be gained with the programme. In addition, the donor-supported provision of higher stipends, medical bags and transportation expenses may have helped to attract more graduates to the PUST programme and Family Medicine in general. It is important to recognize that the forecast presents the *minimum* budget necessary and that it is crucial that MoHSP continues to explore ways to increase its investment in the PUST programme, and overall to increase the attractiveness of Family Medicine as a choice of speciality.

As in any costing study, the results are influenced by the inputs. The change from donor-supported to national budgets depends on the expenses the MoHSP and PMGI are able to take over. The projected scale-up of budgets depends on the targeted number of PUST FDs. Therefore, the results should be interpreted as estimations. The sample size of the PUST evaluation is small, especially the number of newly graduated FDs who completed the conventional one year intern path, and the results might not be representative of all FDs. It unclear how the cost reductions during the transition period might impact the quality of the PUST programme. The current official calculations on the number of FDs needed in Tajikistan are population-ratio based, and might not accurately represent the number of FDs actually needed. Despite this, it can still be concluded that there are not enough FDs in the country, and that the current number of graduating new FDs is not sufficient to resolve the issue. Understandably, training more FDs alone does not resolve the personnel shortage. Other interlinked factors, like salaries, retention of FDs and the ability to attract postgraduates to Family Medicine, also need to be addressed in parallel.

### Recommendations

The following recommendations are made. First, continue international funding for the PUST programme in Family Medicine, to ensure the continuity and structured transition to sustainable national funding. In the next five years, US$ 355,000 is needed from an international donor. Second, MoHSP should commit to increasing national funding for the PUST programme. The minimum five-year investment required from MoHSP is US$ 448,000. Third, MoHSP is advised to commit to scale up PUST to at least to 100 new FDs graduating each year. The level of scaling up alone does not solve the shortage of FDs but is necessary to at least maintain the current coverage of FD positions being filled. Fourth, decrease international financing gradually to prepare for the transition to national funding. Consider making the international support conditional on the increase of national funding and scale-up of the number of new FDs graduating from the PUST programme. And finally, to guarantee sufficient competency levels of new FD graduates, consider using the PUST programme in place of the current one-year internship.

## Conclusions

The PUST programme has been successfully improving the education of FDs in Tajikistan. In order to guarantee the long term supply of adequately trained FDs and address FD shortages, the PUST programme needs to be scaled up, and a transition from international to national funding should begin. International financial support needs to be extended and gradually reduced over the next five years to ensure continuity and to support the transition to sustainable national funding.

## Supplementary Information

**Additional file 1: Table S1** The number of interns, 1st and 2nd-year PUST post-graduates in 2018. **Table S2** Student’s stipends per category of student and by funding source. **Table S3** Additional compensation paid for PUST tutors, trainers and coordinators. **Table S4** PUST related salaries for tutors, trainers and coordinators in 2018. **Table S5** PUST tutor training costs in 2018. **Table S6** Other PUST costs in 2018. **Table S7** The number of family doctors trained, working and needed. **Table S8** Age distribution of working family doctors in 2018. **Table S9** Population growth predictions for Tajikistan 2018–2019.

**Additional file 2: Table S10** Undiscounted annual budgets of PUST in TJS in 2018–2023. *The actual expenses in 2018 and scale-up forecast 2019–2023. **Table S11** Undiscounted annual budgets of PUST in US$ in 2018–2023. *The actual expenses in 2018 and scale-up forecast 2019–2023. **Table S12** Annual discount percentage for the scale-up cost forecast 2019–2023. **Table S13** Discounted annual budgets of PUST in TJS in 2019–2023. **Table S14** Discounted annual budgets of PUST in US$ in 2019–2023.

## Data Availability

All data generated or analysed during this study are included in this published article and its supplementary information files.

## Abbreviations

FD: Family Doctor
FM: Family Medicine
MCQ: Multiple Choice Question
MEP: Medical Education Reform Project
MoHSP: Ministry of Health and Social Protection of the Republic of Tajikistan
OSCE: Objective Structured Clinical Examination
PGMI: Post Graduate Medical Institute
PUST: Post University Specialty Training
SDC: Swiss Agency for Development and Cooperation
