# Supplementary material for: Investment case for two-year post university speciality training in family medicine in Tajikistan: how much is needed for continuing and scaling up the improved education of family doctors?
Source: BMC Health Serv Res. 2020 Dec 9;20:1132. doi: 10.1186/s12913-020-05953-5 (PMC7724868; doi:10.1186/s12913-020-05953-5)
Supplement: Supplementary file 1 — Additional file 1: Table S1 The number of interns, 1st and 2nd-year PUST post-graduates in 2018. Table S2 Student’s stipends per category of student and by funding source. Table S3 Additional compensation paid for PUST tutors, trainers and coordinators. Table S4 PUST related salaries for tutors, trainers and coordinators in 2018. Table S5 PUST tutor training costs in 2018. Table S6 Other PUST costs in 2018. Table S7 The number of family doctors trained, working and needed. Table S8 Age distribution of working family doctors in 2018. Table S9 Population growth predictions for Tajikistan 2018–2019. [file 12913_2020_5953_MOESM1_ESM.docx]

**Additional files**

**Additional file 1**

An overview of the input parameters and their sources used in the economic analysis.

***Table 1*** *The number of interns, 1^st^ and 2^nd^-year PUST post-graduates in 2018.*

| Student’s categories | Students in 2018 | Source |  |
| --- | --- | --- | --- |
| Interns, Tajik State Medical University (TSMU) | 12 | According to Nosirov K., Head of analysis, control and public relations department, Associate Professor, Tajik State Medical University (written communication, February 2019). | |
| Interns, the Post-Graduate Medical Institute (PGMI) | 20 | Gulomova M. O., Information about interns and students studying in 2017-2018 in PGMI. Сведения по интернам и ординаторам обучающихся в 2017-2018 уч. году в ИПОвСЗ. Dushanbe. 2019. 1 p. Report No. 4, 2019 January 18. | |
| 1^st^-year PUST post-graduates PGMI (1^st^-year PUST) | 37 | Gulomova M. O., Information about interns and students studying in 2017-2018 in PGMI. Сведения по интернам и ординаторам обучающихся в 2017-2018 уч. году в ИПОвСЗ. Dushanbe. 2019. 1 p. Report No. 4, 2019 January 18. | |
| 2^nd^-year PUST family doctors PGMI (PUST FDs) | 31 | Gulomova M. O., Information about interns and students studying in 2017-2018 in PGMI. Сведения по интернам и ординаторам обучающихся в 2017-2018 уч. Году в ИПОвСЗ. Dushanbe. 2019. 1 p. Report No. 4, 2019 January 18. | |
| Target PUST FDs in 2023 | 100 | Target of the scale-up | |

***Table 2*** *Student’s stipends per category of student and by funding source.*

| Student’s categories | Number of students | Months | Stipend per month, TJS | Stipends in 2018, TJS | Source |
| --- | --- | --- | --- | --- | --- |
| Interns PGMI | 20 | 11 | 325 Paid by MoHSP | 71,500 | Gulоmova M. O., Information about interns and students studying in 2017-2018 in PGMI. Сведения по интернам и ординаторам обучающихся в 2017-2018 уч. Году в ИПОвСЗ. Dushanbe. 2019. 1 p. Report No. 4, 2019 January 18. |
| 1st and 2nd-year PUST post-graduates PGMI | 68 | 11 | 325  Paid by MoHSP | 243,100 | Gulomova M. O., Information about interns and students studying in 2017-2018 in PGMI. Сведения по интернам и ординаторам обучающихся в 2017-2018 уч. году в ИПОвСЗ. Dushanbe. 2019. 1 p. Report No. 4, 2019 January 18. |
| 1st and 2nd-year PUST post-graduates PGMI | 68 | 11 | 1100 Paid by MEP | 822,800 | Financial records of the budget for support of students on Family Medicine of the 1st and 2nd Year, from Vose, Hamadoni, Rudaki, Hissar, Tursunzade, Penjikent, Devashtich (Ganchi) and Istaravshan. Dushanbe. Medical Education Reform Project (MEP) of the Swiss Tropical and Public Health Institute (Swiss TPH). 2019. |
| Interns TSMU | 11 | 12 | 301  Paid by TSMU | 39,732 | According to Nosirov K., Head of analysis, control and public relations department, Associate Professor, Tajik State Medical University (written communication, February 2019). |

***Table 3*** *Additional compensation paid for PUST tutors, trainers and coordinators.*

| Personnel | Number of personnel | Months | Additional compensation per person per month in 2018, TJS | Compensations in 2018, TJS (calculation) | Source |
| --- | --- | --- | --- | --- | --- |
| Tutors | 32 | 11 | 7,866 | 334,286 | Financial records of the budget for support of students on Family Medicine of the 1st and 2nd Year, from Vose, Hamadoni, Rudaki, Hissar, Tursunzade, Penjikent, Devashtich (Ganchi) and Istaravshan. Dushanbe. MEP, Swiss TPH. 2019. |
| Trainers | 10 | 11 | 12,361 | 250,450 |  |
| Coordinators | 2 | 11 | 22,475 | 77,351 |  |

***Table 4*** *PUST related salaries for tutors, trainers and coordinators in 2018.*

| Personnel | Number of personnel | Months | Gross salary per person per month in 2018, TJS | % of work time used for PUST | PUST related gross salaries in 2018, TJS (calculation) | Source |
| --- | --- | --- | --- | --- | --- | --- |
| Tutors | 32 | 12 | 860 | 25% additional salary for PUST paid by MoHSP | 2,580 | MoHSP. Instruction on the procedure for the appointment and remuneration of employees of state organizations and healthcare institutions of the Republic of Tajikistan. Инструкция по порядку назначения и оплаты труда работников государственных организаций и учреждений здравоохранения Республики Таджикистан. Dushanbe; 2018.108 p. |
| Trainers | 10 | 12 | 1,057 | 100% | 12,684 |  |
| Coordinators | 2 | 12 | 1,350 | 100% | 16,200 |  |

***Table 5*** *PUST tutor training costs in 2018.*

| Tutors trained in 2018 | Costs, TJS | Source |
| --- | --- | --- |
| 32 | 115,212 | Financial records of conducting workshop for tutors Dushanbe in 2018. Dushanbe. MEP, Swiss TPH. 2019. |

***Table 6*** *Other PUST costs in 2018*

| Other categories of costs | Costs in 2018, TJS | Source |
| --- | --- | --- |
| Stationary and gradebook resident for the 1^st^-year PUST post-graduates | 44,533 | Financial records of the budget for support of students on Family Medicine of the 1st Year, from Vose, Hamadoni, Rudaki, Hissar, Tursunzade, Penjikent, Devashtich (Ganchi) and Istaravshan 2018. Dushanbe. MEP, Swiss TPH. 2019. |
| Transportation costs for the 1^st^-year PUST post-graduates | 19,630 | Financial records of the budget for support of students on Family Medicine of the 1st Year, from Vose, Hamadoni, Rudaki, Hissar, Tursunzade, Penjikent, Devashtich (Ganchi) and Istaravshan 2018. Dushanbe. MEP, Swiss TPH. 2019. |
| Doctor bags for the Ordinators- the 1^st^-year PUST post-graduates | 123,025 | Procurement Plan October 2017 - September 2018. Dushanbe. MEP, Swiss TPH. 2019. |
| Stationary for 2^nd^-year PUST- post-graduates | 36,010 | The budget for support of students on Family Medicine of the 2nd Year, from Vose, Hamadoni, Rudaki, Hissar, Tursunzade, Penjikent, Devashtich (Ganchi) and Istaravshan 2018. Dushanbe. MEP, Swiss TPH. 2019. |
| Transportation costs for 2^nd^-year PUST post-graduates | 16,162 | Financial records of the budget for support of students on Family Medicine of the 2nd Year, from Vose, Hamadoni, Rudaki, Hissar, Tursunzade, Penjikent, Devashtich (Ganchi) and Istaravshan 2018. Dushanbe. MEP, Swiss TPH. 2019. |
| Furniture, medical equipment, tuning forks, for the 1^st^- and 2^nd^-year PUST post-graduates | 19,950 | Procurement Plan October 2017 - September 2018. Dushanbe. MEP, Swiss TPH. 2019. |
| Medical literature for the 1^st^- and 2^nd^-year PUST post-graduates | 57,000 | Procurement Plan October 2017 - September 2018. Dushanbe. MEP, Swiss TPH. 2019. |

***Table 7*** *The number of family doctors trained, working and needed.*

| Parameter | Number | Source |
| --- | --- | --- |
| Family doctors trained 1998-2018 | 4,352 | MoHSP. Report of indicators and analysis of the real situation of the healthcare system in 2018. Ҳисоботи муфассали Вазорати тандурустӣ ва ҳифзи иҷтимоии аҳолии Ҷумҳурии Тоҷикистон дар соли 2018. Dushanbe; 2018. p. 9. |
| Family doctors working in 2018 | 3,325 | MoHSP. Report of indicators and analysis of the real situation of the healthcare system in 2018. Ҳисоботи муфассали Вазорати тандурустӣ ва ҳифзи иҷтимоии аҳолии Ҷумҳурии Тоҷикистон дар соли 2018. Dushanbe; 2018. p. 39. |
| Family doctors needed in 2018 | 5,333 | MoHSP. Report of indicators and analysis of the real situation of the healthcare system in 2018. Ҳисоботи муфассали Вазорати тандурустӣ ва ҳифзи иҷтимоии аҳолии Ҷумҳурии Тоҷикистон дар соли 2018. Dushanbe; 2018. p. 9. |

*Table 8: Age distribution of working family doctors in 2018.*

| Age distribution of family doctors | % of family doctors | Source |
| --- | --- | --- |
| Age <30 | 10% | Kiefer S, Lechthaler F, Prytherch H, Yarbaeva S, Kasymova Z, Hojimatova Z et al. Study into staffing needs and supply for primary health care in Tajikistan. Swiss Tropical and Public Health Institute. 2018. 58p. |
| Age 30 < 40 | 24% |  |
| Age 40 < 50 | 22% |  |
| Age 50 < 60 | 36% |  |
| Age 60 < 70 | 9% |  |
| Age 70 and more | 0% |  |

*Table 9 Population growth predictions for Tajikistan 2018-2019.*

| Group | 2018 | 2019 | 2020 | 2021 | 2022 | 2023 | Source |
| --- | --- | --- | --- | --- | --- | --- | --- |
| Urban | 2,501,704 | 2,570,709 | 2,641,682 | 2,718,773 | 2,795,864 | 2,872,956 | Tajikistan Population (2019) - Worldometers [Internet]. Worldometers.info. 2019. Available from: http://www.worldometers.info/world-population/tajikistan-population/ [Accessed 26 Mar. 2019]. |
| Rural | 6,605,507 | 6,721,291 | 6,833,564 | 6,933,495 | 7,033,426 | 7,133,358 |  |
| Total | 9,107,211 | 9,292,000 | 9,475,246 | 9,652,268 | 9,829,291 | 10,006,313 |  |
| Growth rate | 2.08% | 2.03% | 1.97% | 1.87% | 1.83% | 1.80% |  |
