# Supplementary material for: Investment case for two-year post university speciality training in family medicine in Tajikistan: how much is needed for continuing and scaling up the improved education of family doctors?
Source: BMC Health Serv Res. 2020 Dec 9;20:1132. doi: 10.1186/s12913-020-05953-5 (PMC7724868; doi:10.1186/s12913-020-05953-5)
Supplement: Supplementary file 2 — Additional file 2: Table S10 Undiscounted annual budgets of PUST in TJS in 2018–2023. *The actual expenses in 2018 and scale-up forecast 2019–2023. Table S11 Undiscounted annual budgets of PUST in US$ in 2018–2023. *The actual expenses in 2018 and scale-up forecast 2019–2023. Table S12 Annual discount percentage for the scale-up cost forecast 2019–2023. Table S13 Discounted annual budgets of PUST in TJS in 2019–2023. Table S14 Discounted annual budgets of PUST in US$ in 2019–2023. [file 12913_2020_5953_MOESM2_ESM.docx]

**Additional file 2:**

Undiscounted and discounted cost forecasts of the PUST programme for the period 2019-2023.

***Table 10*** *Undiscounted annual budgets of PUST in TJS in 2018-2023. ^*^The actual expenses in 2018 and scale-up forecast 2019-2023.*

| Payer | 2018^*^ | 2019 | 2020 | 2021 | 2022 | 2023 | 2019-2023 |
| --- | --- | --- | --- | --- | --- | --- | --- |
| MoHSP | 485,000 | 609,000 | 748,000 | 858,000 | 966,000 | 1,048,000 | 4,229,000 |
| Donor | 1,675,000 | 1,340,000 | 1,005,000 | 670,000 | 335,000 | 0 | 3,350,000 |
| Total | 2,160,000 | 1,949,000 | 1,753,000 | 1,528,000 | 1,301,000 | 1,048,000 | 7,579,000 |

***Table 11*** *Undiscounted annual budgets of PUST in US$ in 2018-2023. ^*^The actual expenses in 2018 and scale-up forecast 2019-2023.*

| Payer | 2018^*^ | 2019 | 2020 | 2021 | 2022 | 2023 | 2019-2023 |
| --- | --- | --- | --- | --- | --- | --- | --- |
| MoHSP | $51,000 | $65,000 | $79,000 | $91,000 | $102,000 | $111,000 | $448,000 |
| Donor | $177,000 | $142,000 | $106,000 | $71,000 | $35,000 | $0 | $354,000 |
| Total | $228,000 | $207,000 | $185,000 | $162,000 | $137,000 | $111,000 | $802,000 |

***Table 12*** *Annual discount percentage for the scale-up cost forecast 2019-2023.*

|  | % | Source |
| --- | --- | --- |
| Discount % | 6.0% | Inflation, consumer prices (annual %, 2016) - Tajikistan \| Data. The World Bank. (2020). Retrieved 26 June 2020, from https://data.worldbank.org/indicator/FP.CPI.TOTL.ZG?locations=TJ |

***Table 13*** *Discounted annual budgets of PUST in TJS in 2019-2023.*

| Payer | 2019 | 2020 | 2021 | 2022 | 2023 | 2019-2023 |
| --- | --- | --- | --- | --- | --- | --- |
| MoHSP | 646,000 | 840,000 | 1,022,000 | 1,220,000 | 1,402,000 | 5,130,000 |
| Donor | 1,420,000 | 1,129,000 | $798,000 | $423,000 | 0 | 3,770,000 |
| Total | 646,000 | 840,000 | 1,022,000 | 1,220,000 | 1,402,000 | 5,130,000 |

***Table 14*** *Discounted annual budgets of PUST in US$ in 2019-2023.*

| Payer | 2019 | 2020 | 2021 | 2022 | 2023 | 2019-2023 |
| --- | --- | --- | --- | --- | --- | --- |
| MoHSP | $69,000 | $89,000 | $108,000 | $129,000 | $149,000 | $544,000 |
| Donor | $151,000 | $119,000 | $85,000 | $44,000 | $0 | $399,000 |
| Total | $220,000 | $208,000 | $193,000 | $173,000 | $149,000 | $943,000 |
